# Discrepancies in Recommendations on Pharmacokinetic Drug Interactions for Anticancer Medications and Direct Oral Anticoagulants (DOAC): A Comparative Analysis of Different Clinical Decision Support Systems and Sources

**DOI:** 10.3390/ph18071044

**Published:** 2025-07-16

**Authors:** Karolina Nowinski, Roza Chaireti

**Affiliations:** 1 Department of Clinical Pharmacology, Karolinska University Hospital, SE 14186 Stockholm, Sweden; karolina.nowinski@regionstockholm.se; 2 Department of Laboratory Medicine, Karolinska Institutet, SE 17176 Stockholm, Sweden; 3 Department of Hematology, Karolinska University Hospital, SE 14186 Stockholm, Sweden; 4 Department of Medicine Solna, Karolinska Institutet, SE 17176 Stockholm, Sweden; 5 Department of Molecular Medicine and Surgery, Karolinska Institutet, SE 17176 Stockholm, Sweden

**Keywords:** direct oral anticoagulants, cancer, interactions

## Abstract

**Background/objectives:** In some cases of concomitant use of direct oral anticoagulants (DOAC) and certain anticancer medications, pharmacokinetic interactions are expected; however, clinical data is scarce. This report reviews the recommendations on the use of DOAC concurrently with anticancer drugs according to different clinical decision support systems and sources, with a focus on discrepancies. **Methods:** We reviewed the recommendations from the American Heart Association (AHA), European Heart Rhythm Association (EHRA), summary of product characteristics (SPC) in FASS (Swedish medicine information portal), the Swedish clinical decision support system Janusmed, and information from the Food and Drug Administration (FDA) on the concomitant use of apixaban, edoxaban, and rivaroxaban (activated factor X (FXa) inhibitors) and 80 anticancer drugs from 11 categories (240 drug pairs). **Results:** No warnings of expected pharmacokinetic drug interactions between FXa inhibitors and anticancer drugs were found for 155 drug pairs (65%) across all sources. The remaining 35% of drug pairs were flagged as having possible interactions with FXa inhibitors according to at least one source. Discrepancies in the recommendations from the different sources were reported. The reported discrepancies were, for the most part, associated with different assessments of the mechanism and the extent of pharmacokinetic interactions of each anticancer medication. Also, knowledge sources have different approaches to reporting potential interactions, in some cases reporting clinically relevant strictly pharmacokinetic interactions, whereas others include even patient-specific factors. **Conclusions:** The lack of clinical data and different recommendations can make clinical decisions on the concomitant use of DOAC and anticancer drugs difficult. Our compilation is meant to assist clinicians in making decisions based on the available evidence, even if it is scarce.

## 1. Introduction

Direct oral anticoagulants (DOACs), which include apixaban, dabigatran, edoxaban, and rivaroxaban, are commonly prescribed for stroke prophylaxis in patients with atrial fibrillation (AF) and for the prevention and treatment of venous thromboembolism (VTE). Atrial fibrillation is the most common arrhythmia, with a prevalence of more than 10% of individuals over the age of 80 years [1].

The incidence of VTE in Europe and the USA is estimated to be around 1–2 per 1000 person-years [2], but this risk is higher in some populations, such as patients with cancer in whom VTE constitutes a leading cause of morbidity and mortality [3]. Historically, low molecular weight heparin (LMWH) has been the standard treatment for cancer-associated thrombosis (CAT) [4]. However, recent guidelines recommend DOAC, particularly inhibitors of activated factor X (FXa) such as apixaban, edoxaban, and rivaroxaban, as the first-line choice alongside LMWH [5,6,7]. Furthermore, patients with AF may continue taking DOAC for stroke prophylaxis even following a diagnosis of malignancy [8].

Considering the improved outcomes of cancer care leading to an increasing prevalence of cancer among the elderly, including patients using DOAC, and the fact that the risk for most malignancies increases with age [9], the population of cancer patients treated with DOAC is steadily growing. In addition, the number of available cancer medications has increased significantly in recent years.

Pharmacodynamic complications between cancer treatments and DOAC, such as increased risk of bleeding exacerbated by high age and thrombocytopenia induced by cancer treatments, are a serious concern. In addition, kidney failure caused by chemotherapy can cause a reduced elimination of DOAC, which potentially further increases the risk of bleeding. There is also a risk of pharmacokinetic interactions between DOAC and certain anticancer drugs. Apixaban and rivaroxaban are metabolized primarily by CYP3A4, and their excretion is affected by the transport protein p-glycoprotein (P-gp), whereas the elimination of dabigatran and edoxaban is mainly defined by P-gp [10]. Certain anticancer drugs may either induce or inhibit CYP3A/P-gp, which can lead to decreased or increased plasma concentrations of DOAC, thereby increasing the risk of stroke or first-time/recurrent VTE, or increasing the risk of bleeding, respectively [11]. Furthermore, polypharmacy is an important consideration that should not be overlooked when evaluating the risk of drug interactions and complications [12].

Despite the potential clinical relevance and implications of drug interactions between anticancer medications and DOAC, clinical data on such interactions is very scarce. Existing recommendations are mainly based on in vitro data, animal studies, and small patient cohorts. International scientific societies, such as the American Heart Association (AHA) [13] and the European Heart Rhythm Association (EHRA) [14], have published recommendations on the concomitant use of DOAC and cancer medications. Their recommendations are primarily based on information from the summary of product characteristics (SPCs), often extrapolated from in vitro data, comparisons with other drugs with similar pharmacokinetic properties, and expert consensus. This has resulted in discrepancies between information in SPCs and knowledge databases on interactions. Furthermore, differences in genotype and other interindividual characteristics can affect the individual response to each medication [15].

This study was initiated to address the clinical need to provide clinicians with information on interactions between DOACs and anticancer treatments. The purpose was to review and compare information from the summary of product characteristics, a Swedish drug interaction database, and international societies and authorities in order to create a user-friendly guide on drug interactions between DOAC and anticancer treatment for national use (not presented in the article).

## 2. Results

We reviewed 80 anticancer medications, which were grouped into 11 distinct therapeutic groups: antimitotics, antimetabolites, topoisomerase inhibitors, anthracyclines, alkylating agents, platinum-based agents, tyrosine kinase inhibitors, monoclonal antibodies, hormone therapy, immunomodulating agents, and proteasome inhibitors (see Table 1, [14]).

All 80 anticancer drugs were mapped according to pharmacokinetic drug–drug interactions with apixaban, edoxaban, and rivaroxaban, respectively, according to the sources described under Materials and Methods. Thus, 240 drug pairs between each anticancer drug and apixaban, edoxaban, and rivaroxaban, respectively, were reviewed. Full agreement between all reviewed sources was found for 155 drug pairs (65%) of the drug pairs included in the analysis. These drug pairs had no pharmacokinetic interactions identified in any of the sources.

### 2.1. Drug Combinations with No Pharmacokinetic Interactions

No warnings of expected pharmacokinetic drug interactions were found for 155 drug pairs (65%) of anticancer medications and FXa inhibitors consistently between sources.

Specifically, no pharmacokinetic interactions were found between any of the three FXa inhibitors in combination with alkylating agents (bendamustine, busulfan, carmustine, chlorambucil, dacarbazine, melphalan, and temozolomide), antimetabolites (n = 16, see Table 1), platinum-based agents (n = 5, see Table 1), proteasome inhibitors (n = 3, see Table 1), monoclonal antibodies (n = 6, see Table 1), topoisomerase inhibitors (topotecan and irinotecan), anthracyclines (daunorubicin and mitoxantrone), tyrosine kinase inhibitors (gefitinib), or hormone therapies (including flutamide, letrozole, fulvestrant, raloxifene, and leuprorelin). Another 12 drugs (cyclophosphamide, iphosphamide, lomustine, docetaxel paclitaxel, vinorelbine, idarubicine, bicalutamide, dexamethasone, everolimus, sirolimus, and temsirolimus) consistently had no interactions with edoxaban.

### 2.2. Drug Combinations to Avoid or Use with Caution

**Figure 1:**
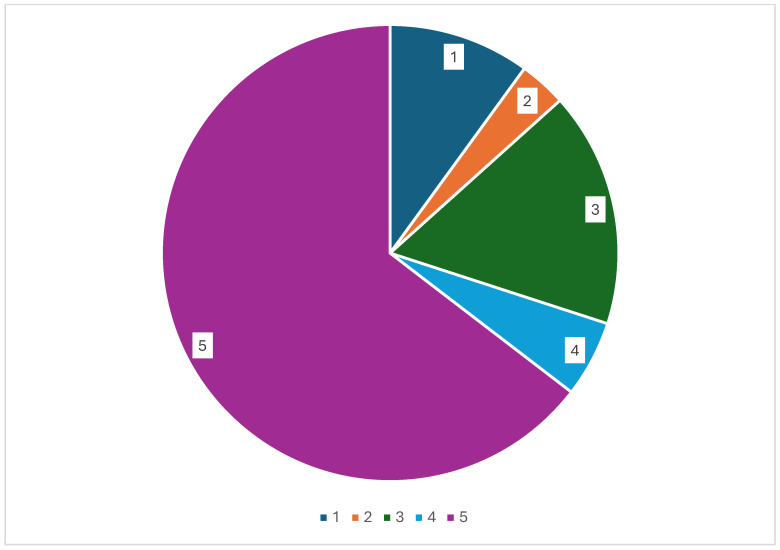
Type of recommendation regarding pharmacokinetic drug interaction (DDI) among 240 drug pairs (n) of anticancer drugs combined with FXa inhibitors. 1 = contraindicated according to EHRA (n = 24), 2 = use with caution/avoid according to EHRA (n = 8), 3 = caution especially in case of polypharmacy or in the presence of at least two bleeding risk factors according to EHRA (n = 40), 4 = no DDI in EHRA but caution in other sources (n = 13), 5 = no DDI in any sources (n = 155).

A total of 85 drug pairs (35%) consisting of 33 anticancer drugs in combination with FXa inhibitors were flagged in one or several sources as contraindicated, to be avoided, or to be used with caution [14,16,17]. Several discrepancies in recommendations or types of interaction were observed between sources (Table 2, Figure 1).

EHRA points out eight anticancer medications (24 drug pairs) as “contraindicated/not advisable” for concomitant treatment with DOAC (Table 2). The greatest variability among sources was observed for drugs where the interactions were reported as caused by strong P-gp induction or inhibition according to EHRA. For example, EHRA and AHA classified doxorubicine as a strong P-gp inducer, and EHRA advises against its concurrent use with FXa inhibitors due to the risk of reduced DOAC plasma levels. However, other sources did not indicate any significant pharmacokinetic drug interactions between doxorubicine and FXa inhibitors. Several tyrosine kinase inhibitors were among those anticancer medications that should not be used according to EHRA. An example was crizotinib, a moderate CYP3A4 inhibitor according to all reviewed sources, which, according to EHRA, was also classified as a strong P-gp inhibitor. The inhibitory effect on P-gp was observed in vitro only according to the SPC.

The recommendation of caution when using an anticancer drug combined with an FXa inhibitor in any of the sources was found for 61 drug pairs. Drugs recommended to “use with caution or avoid” according to EHRA (eight drug pairs) included tacrolimus in combination with FXa inhibitors (due to strong-to-moderate P-gp and mild CYP3A4 inhibition) and cyclosporine in combination with edoxaban (strong-to-moderate P-gp inhibition). There were also warnings (Grade C interactions) for these combinations in Janusmed. Paclitaxel or vemurafenib in combination with apixaban or rivaroxaban were recommended to be used with caution/avoided due to moderate CYP3A4 induction, respectively, while combination with edoxaban was not expected to result in any pharmacokinetic interaction.

A recommendation to use with caution, especially in the case of polypharmacy or in the presence of at least two risk factors for bleeding or interactions, was present for 40 drug pairs according to EHRA. The drug pairs to use with caution due to polypharmacy/bleeding risk factors, grouped according to interaction mechanism, were the following: mild CYP3A4 inhibition (cyclophosphamide, iphosphamide, lomustine, idarubicin, anastrozole, sirolimus, temsirolimus, etoposide, and dasatinib), mild or moderate CYP3A4 induction (docetaxel, vincristine, and dexamethasone), moderate CYP3A4 inhibition (bicalutamide and cyclosporine), and moderate- strong P-gp inhibition in combination with mild CYP3A4 inhibition (tamoxifen, lapatinib, and nilotinib). Edoxaban was the preferred FXa inhibitor for some of these drugs (cyclophosphamide, iphosphamide, lomustine, etoposide, idarubicin, anastrozole, bicalutamide, dexamethasone, docetaxel, paclitaxel, sirolimus, temsirolimus, and vinorelbine) due to the absence of expected pharmacokinetic interactions.

In Janusmed, 28/40 drug pairs (70%) that the EHRA recommends using with caution due to polypharmacy or bleeding risk factors showed no warnings for pharmacokinetic interactions. Janusmed includes clinically relevant pharmacokinetic interactions in a drug pair per se and does not take other factors into consideration, e.g., patient-specific bleeding risk factors. One example of a discrepancy was sirolimus, which was reported by EHRA as a mild CYP3A4 inhibitor. However, the SPC of sirolimus states that the effect on CYP enzymes has been observed in vitro and is not expected to be seen in vivo.

## 3. Discussion

This study examines differences in reported drug–drug interactions (DDIs) between FXa inhibitors (apixaban, edoxaban, and rivaroxaban) and various anticancer drugs according to different sources, including the EHRA guidelines, Janusmed, FDA, and AHA.

Full agreement between sources was found for 65% of the drug pairs, with no identified pharmacokinetic interactions mentioned. Discrepancies between sources regarding reported drug interactions between FXa inhibitors were found for 35% of drug pairs included in this study. These differences were primarily due to differences in reported mechanisms of interaction, such as whether CYPP3A4 or P-gp were induced or inhibited, as well as the strength of this induction or inhibition. Furthermore, EHRA’s recommendations typically emphasized caution, avoidance, or contraindication. In contrast, Janusmed classifies drug–drug interactions according to their clinical relevance and did not categorize any interactions between FXa inhibitors and anticancer medications as contraindicated.

The work included in this report was initiated upon noting discrepancies in reported drug interactions between anticancer medications and FXa inhibitors between different sources, especially when comparing information in international [13,14] recommendations, databases [18], FDA, and SPCs [19]. Considering the increasing usage of DOAC among patients with atrial fibrillation and cancer-associated VTE, the issue of clinically significant pharmacological interactions is of utmost importance for the treating physician. The lack of clinical data and inconsistency between recommendations further complicate the decision-making process.

As mentioned, discrepancies between sources are not unusual. The main reason for this could be that different societies and databases rely on different sources of knowledge and evidence when formulating their recommendations. For instance, the inclusion of results from animal and in vitro studies can alter the foundation of the evidence. In vivo studies are more reliable than evidence from in vitro studies in determining the clinical relevance of P-gp-mediated drug–drug interactions [20].

The reported degree of P-gp inhibition or induction differed between sources in our study and could partially cause the rate of discrepancy in recommendations. This is in line with other work that points out a lack of consistent information on P-gp-mediated DDIs [20]. The authors suggest a systematic protocol based on literature reviews and expert opinion in order to create clinical recommendations for P-gp-related DDIs.

A previous Swedish study comparing the interaction alerts in medication lists of elderly patients found that 41–82% drug pairs resulted in similar alerts in Janusmed compared to Lexicomp^®^, Micromedex^®,^ and Stockley’s Drug Interactions/Checker, respectively [18]. In other studies, the agreement regarding the severity of reported interactions between different sources varies between 20% and 70% [21,22,23]. Given the inconsistencies across these databases, even in the most critical alerts, it is not surprising that our investigation did not reveal a higher level of agreement, especially since clinical data remains limited.

The usage of anticancer medications that inhibit P-gp and/or CYP3A4 can lead to increased plasma concentrations of DOAC, whereas the induction of P-gp and/or CYP3A4 can lead to decreased plasma concentrations of DOAC. The fact that edoxaban was shown to have fewer interactions compared to rivaroxaban and apixaban is likely due to the fact that edoxaban is metabolized by CYP3A4 to a very minimal extent (<4%).

The majority of tyrosine kinase inhibitors (TKIs) have been associated with potentially clinically significant pharmacokinetic interactions when co-administered with FXa inhibitors. However, the underlying mechanism of these interactions and the extent (mild or moderate CYP3A4 inhibition/induction and/or P-gp inhibition) varied across sources, leading to differences in the strength of the resulting clinical recommendations. In patients on TKI, the avoidance or dose reduction of apixaban and the avoidance of rivaroxaban is recommended according to recent European guidelines for atrial fibrillation [24]. Furthermore, an additive effect on hemostasis and therefore risk for bleeding is mentioned for several of the TKIs [17].

An in vitro study evaluating P-gp-mediated DDI between TKIs and apixaban or rivaroxaban using cell cultures showed a risk of drug–drug interaction in vitro between TKIs and DOACs, and that the risk was greater with rivaroxaban compared to apixaban based on differences in the concentration of TKI that inhibits 50% of the transport of DOAC [25]. The results of the study underline the need for in vivo studies in this context.

Following the initiation of TKIs, patients are monitored by analyses of blood samples at regular intervals to check liver and kidney function, as well as hemoglobin, white blood cell counts, and platelet values. If concomitant treatment with DOAC is planned, the first step would be to take a comprehensive bleeding history, e.g., by using a standardized questionnaire such as the ISTH-SCC Bleeding assessment tool (BAT) [26] and considering an investigation in cases of abnormal bleeding history prior to initiating anticoagulant treatment. Following this initiation, rigorous clinical and laboratory follow-up, including testing for DOAC concentration, can be of value. In the case that the results of the aforementioned investigations suggest using a lower or higher dosage of TKIs and/or DOACs outside the frame of current guidelines, a clinical conference is recommended (i.e., consensus). Cooperation between oncologists and coagulation specialists is important to individualize patient treatment.

The determination of DOAC plasma concentrations should be considered, especially at the initiation of treatment, in the case of expected pharmacokinetic drug interactions. The same applies in cases of severely impaired renal function. Following initial controls, if renal function is stable and no new medications that are metabolized by the same mechanisms are introduced, no further frequent controls are needed. In addition to pharmacokinetic interactions, pharmacodynamic interactions, herbal medications, over-the-counter drugs, the effect of body weight/mass and renal function, genetic variations, other risk factors for bleeding and thrombosis, and the indication for the treatment must be taken into account. Importantly, many anticancer medications have independent effects on hemostasis and could increase the risk for bleeding per se or in combination with DOAC. The aforementioned factors were not systematically taken into consideration in the sources we studied and analyzed. Recommendations by the EHRA for certain drug pairs included caution in cases of polypharmacy or in the presence of other risk factors for bleeding. In the daily clinical context, the clinician needs to perform a thorough analysis of all risk factors.

Polypharmacy is most commonly defined as the number of medications an individual is taking and/or the presence of at least one inappropriate medication [27], with the latter often defined according to the Beers criteria [28] or the Medications Appropriateness index [27]. The prevalence of polypharmacy, especially in older patients with cancer, is at least as high as the prevalence in older patients without cancer and naturally increases in the presence of comorbidities [29,30,31]. Considering the risk for DDI, as well as the fact that the risk for adverse events is high in cases of potential drug interactions, different strategies involving periodical medication management interventions and reconciliations, such as multidisciplinary reviewing of medications concerning indication, appropriate dosage (in relation to age, kidney function, and interactions), side effects, compliance, risk for potential DDI, etc., and discontinuation if necessary, are recommended. The team could involve a specialist in clinical pharmacology, in addition to the treating physicians (oncologist, coagulation specialist, general practitioner, etc.) and other appropriate health care personnel [30,31].

Our study is mainly limited by the very issue we are highlighting, namely, the lack of clinical data. It is only by gathering clinical data and performing studies where the effects of DDI on both clinical outcomes and drug concentrations are documented that we can achieve reliable and reproducible results and therefore compose clinical guidelines. Furthermore, the individual response to an interaction is difficult to foresee. Therefore, data on plasma drug concentrations linked to outcome data would be of importance.

The data we have gathered is not sufficient to lead to definite recommendations. However, we have highlighted which combinations could be a risk in any of the sources. Clinicians should be aware that some of these combinations have only been observed in vitro, and therefore, the clinical evidence is still scarce. Extrapolation of the recommendations on managing polypharmacy [30,31] could lead to feasible suggestions involving frequent reviewing of the patient’s medication list (e.g., during a routine follow-up), including dosages and interactions, as well as extra controls in cases of initiation of treatment with new drugs. Specifically, for the interactions between DOAC and anticancer medications, physicians should primarily align patient management with the local/national guidelines, concomitantly with consulting international sources, such as EHRA and other CDSS. In cases of potential interactions and if neither medication can be replaced by an alternative without that risk, surveillance by measuring drug concentrations might be appropriate, as mentioned above.

In conclusion, even if the lack of reliable clinical data makes it difficult to compose guidelines with a high grade of evidence, extrapolated data from experimental studies, pharmacokinetic observations, and interactions between similar drugs can help guide clinicians when making decisions on patient management. Reports such as ours, as well as databases and recommendations [13,14], aim to assist physicians in making clinical decisions, rather than being the sole factor to weigh in. The discrepancies between different knowledge sources underline the importance of multidisciplinary collaboration when assessing the risk of drug–drug interactions.

## 4. Materials and Methods

### 4.1. Clinical Decision Support Systems and Recommendations

The anticancer medications used in the article were chosen and grouped according to the list utilized in the EHRA guidelines [14]. This choice was based on the widespread recognition of the guidelines [14]. Another two drugs were added to the review (ibrutinib and azathioprine). The DOACs included in our review were the FXa inhibitors approved in Sweden, i.e., apixaban, edoxaban, and rivaroxaban, which are also the FXa inhibitors most widely used internationally. Dabigatran, which is an inhibitor of activated coagulation factor II (thrombin), was not reviewed since it is not included in guidelines for the treatment and prevention of cancer-associated thrombosis and is thus the least used DOAC in this setting. Additionally, although apixaban, edoxaban, and rivaroxaban have similar mechanisms of action [32], their metabolisms are varied enough to warrant separate reviewing and lead, in many cases, to different recommendations.

In addition to EHRA [14], we reviewed the recommendations issued by the American Heart Association (AHA) [13], Janusmed Interactions and Risk profile (Janusmed) [16], and information from the summary of product characteristics (SPC) in FASS (Swedish medicines information portal) [17].

Janusmed is a clinical decision support system (CDSS) that has been available online for all prescribers in Sweden since autumn 2022 (and in Region Stockholm, i.e., the regional public body responsible for healthcare within Stockholm County, since 2017) and is embedded in the electronic healthcare record system in some regions in Sweden [33]. It is a non-commercial CDSS designed for the pharmacological risk assessment of a patient’s complete medication list, providing information on pharmacokinetic drug interactions. The pharmacokinetic interactions are graded A–D according to their clinical significance and 0–4 according to the grade of available documentation. The scope of Janusmed is to include clinically relevant pharmacokinetic interactions, as documented in the scientific literature or the SPCs, as well as those expected due to the specific metabolism and elimination of individual drugs [33,34]. Janusmed also provides a risk profile based on nine common or serious adverse events, such as, among others, bleeding risk [34,35].

The general principles of the EHRA classification for drug–drug interactions are that strong CYP3A4 and/or P-gp inducers or inhibitors should not be used, and moderate CYP3A4 or P-gp inducers should be used with caution or avoided. Anticancer medications classified as mild CYP3A4 and/or P-gp inducers or inhibitors were recommended to be used with caution, especially in the presence of polypharmacy or at least two factors that increase bleeding risk (e.g., concomitant antiplatelet drug use, severe frailty, history, or predisposition to bleeding).

### 4.2. Data Collection

The recommendations regarding pharmacokinetic drug interactions between individual anticancer medications and FXa inhibitors from the previously mentioned sources [13,14,16,17] were compiled into a document. The study data included (i) the type and grade of pharmacokinetic interactions, (ii) the mechanism (induction/inhibition of P-gp and/or CYP3A4), and (iii) recommendations on management (if available). Even data on thromboembolic and bleeding adverse side effects from the SPCs [17] and bleeding risk profile from Janusmed [16] were gathered but are not reported in this manuscript. Data on whether the anticancer medication was classified as an inducer or inhibitor of P-gp or CYP3A4 was gathered from all sources [13,14,16,17], as well as from the FDA [19].

### 4.3. Management and Analysis of Data

Following the collection of all data, we reviewed all recommendations to identify discrepancies in the reported status of each anticancer medication as a strong inhibitor/inducer of CYP3A4 and/or P-gp and whether a recommendation to avoid concomitant use of the anticancer drug and DOAC or not was consistent across all sources. In cases of discrepancies, we performed an individual evaluation for each drug interaction and compiled a list of suggestions on clinical management. If nothing else is stated, the reported results on the recommendations from the various sources refer to all three FXa inhibitors investigated. In cases of discrepancies in the recommendations, both authors reviewed the information offered by all sources and conferred before reaching a conclusion.

### 4.4. Statistics

Data analysis and descriptive statistics were performed using Microsoft Excel (version 2024, Microsoft Corporation, Redmond, WA, USA).

## Figures and Tables

**Table 1:** Anticancer medications included in the study.

Drug Group	Medications
Antimitotic agents	docetaxel, paclitaxel, vinblastine, vincristine, vinorelbine
Antimetabolites	azacitidine, azathioprine, capecitabine, cladribine, clofarabine cytarabine, decitabine, fludarabine, fluorouracil, gemcitabine, mercaptopurine, methotrexate, nelarabine, pemetrexed, tegafur, thioguanine
Topoisomeras inhibitors	etoposide, irinotecan, topotecan
Anthracyclines	daunorubicin, doxorubicin, idarubicin, mitoxantrone
Alkylating agents	bendamustine, busulfan, carmustine, cyclophosphamide, dacarbazine, iphosphamide, klorambucile, lomustine, melphalan, temozolomide
Platinum-based agents	bleomycin, carboplatin, cisplatin, mitomycin C, oxaliplatin
Tyrosine kinase inhibitors	crizotinib, dasatinib, erlotinib, gefitinib, imatinib, ibrutinib, lapatinib, nilotinib, sunitinib, vandetanib, vemurafenib
Monoclonal antibodies	alemtuzumab, bevacizumab, brentuximab, cetuximab, rituximab, trastuzumab
Hormone treatment	abiraterone, anastrozole, bicalutamide, enzalutamide, flutamide, fulvestrant, letrozole, leuprorelin, mitotane, raloxifene, tamoxifen
Immunomodulating agents	cyclosporine, dexamethasone, everolimus, sirolimus, tacrolimus, temsirolimus
Proteasome inhibitors	bortezomib, ixazomib, karfilzomib

**Table 2:** Anticancer medications that, according to EHRA, are contraindicated/not advisable (n = 8) and the corresponding recommendations provided in Janusmed Interactions and Risk Profile. The recommendations according to EHRA and Janusmed concern apixaban, edoxaban, and rivaroxaban unless otherwise indicated.

	Mechanism According to EHRA	Mechanism According to Janusmed	EHRA-Recommendation	Janusmed-Recommendation
Antimitotic agent				
Vinblastine	Strong P-gp induction	N/A	Contraindicated/not advisable	No DDI
Anthracyclines				
Doxorubicin	Strong P-gp induction, mild CYP3A4 inhibition	N/A	Contraindicated/not advisable	No DDI
Tyrosine kinase inhibitors				
Imatinib	Moderate CYP3A4 inhibition, strong P-gp inhibition	Moderate CYP3A4 inhibition and/or P-gp inhibition	Contraindicated/not advisable	Caution Grade C
Crizotinib	Moderate CYP3A4 inhibition, strong P-gp inhibition	Moderate CYP3A4 inhibition	Contraindicated/not advisable	Caution for apixaban and rivaroxaban (Grade B), no DDI for edoxaban
Vandetanib	Strong P-gp inhibition	N/A	Contraindicated/not advisable	No DDI
Sunitinib	Strong P-gp inhibitor	N/A	Contraindicated/not advisable	Caution (Grade C) Additive effect on haemostasis but no DDI
Hormone treatment				
Abiraterone	Moderate CYP3A4 inhibition, strong P-gp inhibition	N/A	Contraindicated/not advisable	No DDI
Enzalutamide	Strong CYP3A4 induction, strong P-gp inhibition	Strong CYP3A4 induction	Contraindicated/not advisable	Caution (Grade C) for apixaban and rivaroxaban, Grade B for edoxaban

DDI = pharmacokinetic drug–drug interaction, N/A = not applicable. Janusmed grade B interaction: clinical significance unknown and/or varies. Grade C interaction is defined as a clinically relevant interaction that can be managed.

## Data Availability

Data is available upon request.

## Abbreviations

The following abbreviations are used in this manuscript:

AF	Atrial fibrillation
AHA	American Heart Association
CAT	Cancer-associated Thrombosis
CDSS	Clinical Decision Support System
DOAC	Direct Oral Anticoagulants
EHRA	European Heart Rhythm Association
FXa	Activated factor X
FDA	Food and Drug Administration
LMWH	Low Molecular Weight Heparin
P-gp	P-glycoprotein
SPC	Summary of Product Characteristics
VTE	Venous Thromboembolism
